# Profiles of obstetric interventions during COVID-19 and advocacy for Sustainable Development Goals

**DOI:** 10.1590/0034-7167-2024-0135

**Published:** 2025-09-01

**Authors:** Thalita Beatriz Santos Maciel, Mery Natali Silva Abreu, Thales Philipe Rodrigues da Silva, Ana Paula Vieira Faria, Rafaela Siqueira Costa Schreck, Carolina Machado Moreira, Bruna Figueiredo Manzo, Fernanda Penido Matozinhos

**Affiliations:** I Universidade Federal de Minas Gerais. Belo Horizonte, Minas Gerais, Brazil; II Universidade Federal de São Paulo. São Paulo, São Paulo, Brazil

**Keywords:** Obstetric Interventions, Birth Assistance, COVID-19, Nursing, Sustainable Development, Intervenciones Obstétricas, Asistencia al Parto, COVID-19, Enfermería, Desenvolvimiento Sustentable

## Abstract

**Objectives:**

to assess the profiles of obstetric interventions during the COVID-19 pandemic in public hospitals in Belo Horizonte, Minas Gerais, Brazil.

**Methods:**

an observational study, with a cross-sectional design, nested in a cohort, including 1,717 postpartum women. To identify the profiles of obstetric practices, the Principal Component Analysis technique was used.

**Results:**

divergent profiles of coexistence of obstetric interventions were generated in women with similar obstetric profiles. Given that women belonging to the profiles formed tended to receive the same set of interventions, it was concluded that the results formed three types of profile, which may have been influenced by the context of COVID-19.

**Conclusions:**

the findings of this study demonstrate the need to improve care during labor and birth, aiming to promote autonomy, leading role and well-being of postpartum women. Such a procedure can be decisive in maternal and child outcomes monitored by the Sustainable Development Goals.

## RESUMEN

**Objetivos:**

Evaluar los perfiles de las intervenciones obstétricas durante la pandemia de COVID-19 en hospitales públicos de Belo Horizonte, Minas Gerais, Brasil.

**Métodos:**

estudio observacional, con diseño transversal, basado en una cohorte, que incluyó a 1.717 puérperas. Para identificar los perfiles de las prácticas obstétricas se utiliza la técnica del Análisis de Componentes Principales.

**Resultados:**

se generaron perfiles divergentes de convivencia de intervenciones obstétricas en mujeres con perfiles obstétricos similares. Considerando que las mujeres pertenecientes a los perfiles formados tenderán a recibir el mismo conjunto de intervenciones, concluyo que los resultados formarán tres tipos de perfiles, que pueden verse influenciados por el contexto de COVID-19.

**Conclusiones:**

Los aspectos de este estudio demuestran la necesidad de mejorar la asistencia al parto y al parto, con el objetivo de promover la autonomía, el protagonismo y el bienestar de la mujer en trabajo de parto. Un procedimiento de este tipo puede ser decisivo en los resultados maternos e infantiles monitoreados por el programa de Objetivos de Desarrollo Sostenible.

## INTRODUCTION

Models of care for childbirth and birth have undergone numerous transformations throughout the world over the years. Childbirth has evolved from a moment of exclusive female experience, carried out in the home environment and characterized, primarily, as a physiological event, to an institutionalized process, recognized as a pathological occurrence, marked by medicalization^(1)^.

Medicalization transforms non-medical issues into medical ones, imposing the standardization of bodies and social and sexual practices. This practice alters aspects of the daily life of women’s bodies, such as menstruation, pregnancy and childbirth, for social medical purposes, and they come to be seen as an object and knowledge exclusive to medicine^(2)^.

In recent years, labor assistance has undergone important changes around the world, resulting in the increased use of various practices aimed at regulating and monitoring the physiological process of birth^(3)^. The main objective of such practices would be to improve perinatal outcomes. However, it is important to note that interventions carried out without clinical indications negatively compromise women’s experience of childbirth^(3)^.

Intervention practices during labor and birth, such as the use of forceps, cesarean section and infusion of oxytocin-type medications, performed with clinical indications have contributed to reducing maternal and neonatal morbidity and mortality. However, in the wake of this success, the idea of extending their benefits to all births, to prevent negative outcomes, has spread, leading to the indiscriminate and routine use of such practices^(4)^.

From the perspective of risk control, interventionist attitudes of medicalization are shaped in relation to the female body during childbirth. Arguments in favor of safety and prevention of complications are configured as instruments capable of legitimizing the notion of the imperfect functioning of postpartum women’s body, which requires preventive and curative practices and technologies to avoid unfavorable outcomes^(5)^.

In Brazil, the increase in unnecessary interventions is related to medicalization of obstetric care, leading to high rates of cesarean sections without clinical indication. Today, contrary to the reference rate adjusted for the Brazilian population, which is between 25% and 30%, the country has a cesarean section rate of 55% in the public health system and 86% in the private health care sector, positioning itself as one of the world leaders in this surgery^(6)^.

The World Health Organization (WHO), in the context of the document “Good practices in care during normal labor and childbirth”^(7)^, has been recommending, since 1996, that the use of interventions be based on scientific evidence, defining four categories: a) Practices that are clearly useful and should be encouraged; b) Practices that are clearly harmful or ineffective and should be eliminated; c) Practices used inappropriately during labor; and d) Practices that require studies for scientific proof.

In 2018, the WHO updated the document proposed in 1996, starting to be guided by the Sustainable Development Goals (SDGs), from a women’s health perspective to reduce maternal mortality, with a focus on adopting respectful maternity care, not only to avoid complications during childbirth, but also to make the experience of giving birth positive, with better physical, mental and psychological results, both for the mother and the baby and the family^(3)^.

A study conducted in Brazil showed that medicalized care during labor and birth is characterized mainly by the significant number of cesarean sections and the routine use of other types of intervention, such as trichotomy, episiotomy, amniotomy and lithotomy position at birth. The study also highlighted that such interventions are recognized as clearly harmful practices, which is why they should be eliminated in childbirth care or used with restrictions, as they can influence the prevalence of puerperal complications^(8)^.

Authors have shown that obstetric nurses’ or midwives’ work contributes to the greater adoption of good care practices at childbirth. The humanization actions of these professionals are seen as resistance to medicalization of childbirth, based on confronting interventionist medical practices, defending the physiology of childbirth and comprehensive care^(9)^. Additionally, it is noted that obstetric nurses’ care seeks to promote relationships of encounter with the other (the woman) and technical support, which contributes to the participation of women and the reduction of unnecessary interventions^(10)^.

The COVID-19 pandemic has brought about changes in the context of childbirth and birth in Brazil, as it has required adaptations in health practices. Preventing this disease has become the central objective, since little was known about the consequences of infection during pregnancy. From this perspective, the “mother-child” dyad may have undergone more obstetric interventions^(11)^.

Due to the various physiological changes present during pregnancy, especially in the immune and respiratory systems, pregnant women were included in the COVID-19 risk group, along with postpartum women, older adults and people with chronic diseases, as they are at higher risk if infected^(12)^.

Studies have shown high rates of maternal morbidity and mortality, including preterm birth, preeclampsia, placental abruption, pulmonary edema, and the need for mechanical ventilation as obstetric outcomes resulting from COVID-19 infection^(13,14)^.

This study was guided by the following research question: what are the profiles of obstetric interventions identified during the COVID-19 pandemic?

## OBJECTIVES

To assess the profiles of obstetric interventions performed during the COVID-19 pandemic in public hospitals in Belo Horizonte, Minas Gerais, Brazil.

## METHODS

### Ethical aspects

The study was approved by the *Universidade Federal de Minas Gerais* Research Ethics Committee as part of a larger study, “*Parto e aleitamento materno em filhos de mães infectadas por SARS-CoV-2*”. Patient consent was waived because data collection was based on medical records.

### Study design, period and location

This is an observational study with a cross-sectional design, nested in a cohort based on data from the study “Childbirth and breastfeeding in children of mothers infected with SARS-CoV-2”. It was carried out in three public maternity hospitals that are references and exclusive for the care of the Brazilian Health System (In Portuguese, *Sistema Único de Saúde* - SUS), with the presence of a multidisciplinary team, including obstetric nursing professionals, in the city of Belo Horizonte, Minas Gerais, Brazil, from 2020 to 2022.

The EQUATOR network guidelines were adopted through the STrengthening the Reporting of OBservational studies in Epidemiology (STROBE) tool.

### Population and inclusion and exclusion criteria

The sample was constituted by means of a random draw among women who gave birth in the respective hospitals during the three months of high incidence of COVID-19 (May, June and July) of 2020 in Brazil.

This study included all postpartum women with hospital births and singleton pregnancies who gave birth to live newborns weighing more than 500 grams at birth and who were admitted to the three selected maternity hospitals at the time of delivery.

For sample calculation, a cohort study design was considered. The distribution of the number of pregnant women per participating maternity hospital respected the proportion of the total number of births in each selected maternity hospital. A total of 1,717 postpartum women were selected for this study.

Since this is a cross-sectional study nested within a cohort, data collection comprised three distinct and independent stages. The first consisted of retrospective collection from electronic hospital records. The second and third stages consisted of telephone collection, divided into two stages: the first, between 6 and 12 months postpartum, and the second, between 12 and 24 months postpartum. Pregnant women were accessed at different times, and telephone contact, carried out by trained researchers, considered at least five attempts. In cases where pregnant women refused to participate in the study or attempts to contact them were unsuccessful, they were excluded/replaced. The data collection sample consisted of 480 pregnant women who responded to telephone contact during the data collection process. For this reason, the sample profile presented different values from the sample number for some variables.

### Study protocol

Data were collected from hospital records during the period of greatest COVID-19 infection, between 2020 and 2022, using a semi-structured questionnaire adapted from the research “*Nascimento em Belo Horizonte: trabalho de parto e Pesquisa de Nascimento*”^(15)^.

The study variables were selected based on an analytical model on the topic and the 2018 update of the “Intrapartum Care for a Positive Childbirth Experience” WHO guideline^(3)^.

The variables were divided into three categories (Chart 1).

**Chart 1:** Variables selected for the study

Sociodemographic and health factors	Obstetric factors	Obstetric practices
Age. Education. Skin color. Paid employment. COVID-19 infection.	Number of prenatal consultations. Primigravida. Gestational age at delivery. Presence of an obstetric nurse. Companion during delivery. Mode of delivery.	Intravenous hydration. Offering diet. Freedom of movement. Use of partograph. Non-pharmacological methods for pain relief. Enema. Trichotomy. Lying on the back with legs raised. Kristeller maneuver. Amniotomy. Oxytocin infusion. Analgesia. Episiotomy. Peripheral venous access puncture. Use of forceps.

### Analysis of results, and statistics

Initially, the variables were presented in absolute and relative frequencies (%), with the respective 95% Confidence Intervals (CI), when categorical. Regarding the numerical variables, after verifying asymmetry, based on the Shapiro-Wilk test, the results were presented through median and interquartile range (IQR).

To identify the profiles of obstetric practices, the Principal Component Analysis (PCA) technique was used. This is an exploratory analytical method that condenses the information contained in the original observed variables into a smaller number of variables, with minimal loss of information. PCA is a statistical multivariate analysis technique that linearly transforms an original set of variables that were initially correlated with each other into a substantially smaller set of uncorrelated variables that contain most of the information in the original set^(16)^.

The variables included in the PCA were intravenous hydration, diet provision, freedom of movement, use of partograph, non-pharmacological methods for pain relief, enema, trichotomy, supine position with legs raised, Kristeller maneuver, amniotomy, oxytocin infusion, analgesia, episiotomy, peripheral venous access puncture and use of forceps.

The variables intravenous hydration, enema, trichotomy, Kristeller maneuver, amniotomy, and use of forceps did not achieve satisfactory factor loadings, which is why they were removed from the model. The Kaiser-Mayer-Olkin (KMO) coefficient was estimated as a measure of PCA adequacy, with values between 0.5 and 1.0 considered acceptable for this index. Eigenvalues greater than 1 were considered in the definition of the number of components, based on the scree plot graph. The component structure was obtained from indicators that presented factor loadings greater than 0.3 or less than -0.3. Factors with high factor loading (above 0.30, based on the adopted criterion) in more than one component were considered in that component with the highest factor loading.

The data were analyzed using Stata version 16.0.

## RESULTS

This study assessed 1,717 patients. The median age was 31 years (IQR: 26-36). The most prevalent level of education was complete high school (59.4%), and the predominant self-reported skin color was brown (57.2%). Finally, 311 patients (64.8%) had a paid job and 34 (4.3%) presented some sign or symptom of COVID-19 (Table 1).

**Table 1:** Sample profile, Belo Horizonte, Minas Gerais, Brazil, 2023

Variable	Median and IQR^*^			
Age	31(26 -36 )			
Education	n	%	IC 95%^**^	
Higher education	68	14.1	11.3	17.5
Complete high school	285	59.3	54.9	63.6
Complete elementary school	102	21.2	17.8	25.1
Complete primary school	25	5.21	3.53	7.60
Skin color	n	%	IC 95%	
White	88	18.3	15.1	22.0
Black	102	21.2	17.8	25.1
Brown	276	57.5	53.0	61.8
Yellow	12	2.5	1.4	4.3
Indigenous	2	0.4	0.1	1.6
Paid work	n	%	IC 95%	
Yes	311	64.7	60.3	68.9
No	169	35.2	31.0	39.6
Suspected or confirmed COVID				
No	749	95.6	93.9	96.8
Yes	34	4.3	3.1	6.0

* IQR - interquartile range;

** 95% CI - 95% Confidence Interval.

Regarding gynecological and obstetric variables, 78.3% of women had six or more prenatal consultations and 62.6% were not primiparous. The median gestational age at delivery was 39 weeks. Most women were monitored by obstetric nurses (91.8%), and 90.4% had a companion during delivery. The most common type of labor observed in the sample was spontaneous (51.8%) (Table 2).

**Table 2:** Characterization of gynecological-obstetric variables, Belo Horizonte, Minas Gerais, Brazil, 2023

	n	%	95% CI^*^	
Number of prenatal consultations				
≥6	956	78.3	75.9	80.5
<6	264	21.6	19.4	24.0
Primigravida				
Yes	642	37.3	35.1	39.7
No	1.075	62.6	60.2	64.8
Gestational age at delivery^**^	39(38 - 40)			
Presence of a professional obstetric nurse				
No	104	8.18	6.79	9.82
Yes	1167	91.8	90.1	93.2
Presence of a companion during childbirth				
Yes	1259	90.4	88.7	91.8
No	133	9.5	8.1	11.2
Labor				
Spontaneous	746	51.8	49.2	54.4
Unsuccessfully induced - referred for cesarean section	173	12.0	10.4	13.8
Successfully induced	450	31.2	28.9	33.7
Did not go into labor - cesarean section	69	4.8	3.8	6.0

* 95% CI - 95% Confidence Interval;

** Median and interquartile range.

Table 3 presents descriptive statistics on obstetric practices used during labor and delivery. Regarding obstetric practices considered useful and that should be encouraged, 8.8% included offering a diet, 91.3%, freedom of movement, 60.50%, use of a partograph during labor, and 66.3%, use of non-pharmacological methods for pain relief.

**Table 3:** Characterization of obstetric practices during childbirth, Belo Horizonte, Minas Gerais, Brazil, 2023

	n	%	95% CI^*^	
Obstetric practices considered useful and which should be encouraged				
Diet offer				
No	14	1.1	0.6	1.9
Yes	1,176	98.8	98.0	99.3
Freedom of movement and movement				
No	103	8.6	7.1	10.3
Yes	1,091	91.3	89.6	92.8
Use of partograph				
No	491	39.5	36.8	42.2
Yes	752	60.5	57.7	63.1
Non-pharmacological methods for pain relief				
No	406	33.6	30.9	36.3
Yes	802	66.3	63.6	69.0
Obstetric practices considered ineffective and which should be eliminated				
Enema				
No	1145	99.9	99.3	99.9
Yes	1	0.09	0.01	0.61
Trichotomy				
No	1149	99.6	99.0	99.8
Yes	4	0.3	0.1	0.9
Lying position on back with legs raised during labor				
No	1070	92	90.3	93.5
Yes	92	7.9	6.4	9.6
Kristeller maneuver				
No	1186	99.3	98.6	99.6
Yes	8	0.6	0.3	1.3
Obstetric practices often used inappropriately				
Venous hydration				
No	1073	91.4	89.7	92.9
Yes	100	8.5	7.0	10.2
Oxytocin infusion				
No	881	71.5	68.9	74
Yes	350	28.4	25.9	31
Analgesia				
No	887	71.5	69.0	74.0
Yes	352	28.4	25.9	30.9
Episiotomy				
No	1166	98.3	97.3	98.9
Yes	20	1.6	1.0	2.6
Access				
No	720	62.6	59.7	65.3
Yes	430	37.3	34.6	40.2

* *95% CI - 95% Confidence Interval.*

As for practices considered ineffective and that should be eliminated, enema was observed in only 0.09% and trichotomy was performed in 0.35%. Concerning the birth position, 7.91% gave birth in lithotomy position. The Kristeller maneuver was used in 0.67% of women.

In relation to obstetric practices frequently used inappropriately, 8.5% received intravenous hydration, 28.3% had oxytocin infusion, 28.4% used analgesia during labor and delivery, 98.3% did not undergo episiotomy and 62.6% did not use peripheral venous access (Table 3).

Table 4 shows the results of PCA. From the adopted cut-off point, based on the screen plot, for eigenvalues greater than 1, three components were extracted with a total explained variance of 47.73%.

**Table 4:** Factor loadings of the components of Principal Component Analysis, Belo Horizonte, Minas Gerais, Brazil, 2023

Variable	Component 1	Component 2	Component 3
Diet offer			0.5150
Freedom of movement and movement			0.5762
Use of partograph		-0.4753	
Non-pharmacological methods for pain relief		-0.3943	
Lying position on back with legs raised during labor		0.5836	
Oxytocin infusion	0.5722		
Analgesia	0.5226		
Episiotomy		0.4399	
Peripheral venous access	0.5291	0.2033	
Eigenvalue	1.92	1.28	1.08
Explained variance (%)	21.39	35.66	47.73
Overall KMO	0.623		

*Note: to facilitate understanding, factor loadings lower than 0.3 were eliminated and only the largest loading was considered, when it appeared in two components.*

Component 1 was composed of women who underwent oxytocin infusion, used analgesia and used peripheral venous access.

Component 2 was composed of pregnant women who did not use a partograph, did not receive non-pharmacological methods for pain relief, had their children in the lithotomy position and underwent episiotomy.

The third component consisted of pregnant women who were offered a diet and freedom of movement during labor and delivery. The analysis achieved a satisfactory KMO (above 0.5).

## DISCUSSION

This study generated divergent profiles of coexistence of obstetric interventions in women with similar obstetric profiles. Considering that women who fit into the profiles formed tended to receive the same set of interventions, it is necessary first to understand the benefits and harms of each intervention, particularly the influence of their coexistence on the results obtained, as well as the pandemic context, considering the possibility of having impacted the change in these profiles identified in the analysis of results.

The sociodemographic profile of the population assessed was characterized as young, brown women who completed elementary school and who worked outside the home during the pandemic. Their infection rate was considered low, with suspected or confirmed COVID-19 infection of 2%. However, this may be an underestimate, since the number of COVID-19 tests in Brazil was low and there was a significant delay in the release of results. The tests offered took, on average, seven business days to issue the results, increasing the chances of the disease becoming critical and delaying the timely care strategy. These were important problems in accessing adequate COVID-19 care for pregnant and postpartum women^(17)^.

This study found that the mean age was 30.8 years. This finding is similar to a similar study conducted in Germany, in which the mean maternal age of women who gave birth was 33 years. Moreover, the age at which women have been giving birth in recent years has been increasing, and the proportion of German women who gave birth over the age of 35 increased by 37% in 2018^(18)^.

The mean number of prenatal consultations identified in this study was 7.82, a value compatible with that recommended by the Ministry of Health, which recommends that pregnant women with normal risk have at least six prenatal consultations (one in the first trimester of pregnancy, two in the second and three in the third), with the first consultation ideally taking place in the first trimester and monthly consultations being carried out until the 34^th^ week^(19)^.

Prenatal consultations are extremely important to inform women about the labor and delivery process, and should provide information that allows them to make autonomous choices so that, armed with information, they are less vulnerable to interventions. In Brazil, within the scope of SUS, the Stork Network stands out, with the proposal of actions structured based on four components: prenatal care; labor and birth; puerperium and comprehensive child health care; and logistics system, which refers to health transport and regulation. In this context, the prenatal axis is highlighted as a fundamental element to guarantee quality, safe and humanized care for all women^(20)^, including critical epidemiological moments, such as COVID-19.

The first profile, formed by interventions frequently used inappropriately in labor assistance, includes postpartum women who underwent oxytocin infusion, used analgesia and peripheral venous access puncture, and underwent episiotomy.

Interventions performed during labor and birth care must be carried out judiciously and in situations where there is a clinical indication of real need. However, such interventions often occur arbitrarily, affecting many women who are treated in hospitals in the country, including the indiscriminate use of oxytocin^(3)^. Oxytocin is a hormone produced by the hypothalamus and stored in the posterior pituitary gland, whose action is central in labor, as it is responsible for stimulating uterine contractions^(21)^. Although it is not recommended by the WHO, and is part of a group of practices that are used inappropriately in obstetric care, synthetic oxytocin is routinely used in many Brazilian maternity hospitals, with the aim of increasing and accelerating contractions^(21)^. The data found by this research revealed the use of oxytocin for 28.43% of women assisted in the institutions investigated, a considerable value, but below the national average of 40% for the administration of this hormone among Brazilian postpartum women^(22)^.

The practice of episiotomy is an obstetric intervention that consists of a cut made in the perineal region, which enlarges the area where the baby can exit, speeding up the birth process, and should not be used the usual way^(3)^. Since it is a surgical procedure, the woman must be informed and authorized before it is performed, and the possible risks and benefits must be pointed out. This intervention has often been used routinely in most Brazilian institutions without adequate information for women, despite its recommendation covering only 10 to 15% of cases^(23)^. However, this research identified that 1.69% of women underwent episiotomy during childbirth care, therefore revealing a scenario closer to that recommended by the WHO so that this practice should be discouraged^(3)^.

This study concluded that venipuncture affected 37% of postpartum women. In most cases, venipuncture is associated with the use of synthetic oxytocin for induction and acceleration or correction of changes in the progression of labor, and is considered, in most cases, an unnecessary intervention^(24)^.

The second profile of this work took into consideration the use of obstetric practices that are clearly harmful and should be abolished in obstetric care, considering women who did not use a partograph, did not receive non-pharmacological methods for pain relief and gave birth in the lithotomy position.

Partograph is a tool developed to improve communication between women and health professionals. It was found that 60.50% of women in the institutions investigated used the partograph during labor, a higher proportion than that found in the literature^(25)^, an aspect that demonstrates the need to achieve better adherence to the use of a partograph in obstetric care by health professionals. The use of this tool makes up the profile of practices that are useful and benefit women in the childbirth process.

The use of analgesia during labor is common and has benefits for the woman in labor, making the process of giving birth less painful. Therefore, although analgesia is characterized as an inappropriately used practice, in vaginal delivery its use should be guaranteed and women should be provided with whatever they request, with assessment and monitoring of maternal and fetal well-being. There are options for local analgesia and non-pharmacological methods for pain relief. A study found that the use of local analgesia was lower in proportions of pregnant women who were monitored by obstetric nursing professionals^(26)^.

Non-pharmacological methods are an option to replace analgesia during labor and delivery and to help postpartum women deal with their pain complaints^(27)^. This study found that non-pharmacological methods of pain relief were used by 66.39% of women, with showers, baths, massages, use of balls, stools and birthing stools among the most commonly used methods based on the literature. Another study showed that most women who used non-pharmacological methods of pain relief had vaginal deliveries^(28)^, contributing to the reduction of anxiety and pain, disorders listed as the main causes of the increase in the number of elective cesarean sections^(29)^.

The third profile highlighted practices that are useful and benefit women during the birth process, considering women who were given free access to a diet and freedom of movement. This research showed that 91.37% of women had freedom of movement during the birth process, which, during labor, helps with the progression of labor, helps to cope with pain and reduces the risk of episiotomy^(28)^, which is why it is indicated as a useful obstetric practice that should be recommended^(3)^. During the pandemic, most birthing positions were encouraged, with preference given to vertical births. However, there was guidance that water births should be avoided due to the presence of viruses in feces, with a high potential for contamination^(29)^.

A qualitative study carried out with the purpose of assessing the repercussions of the pandemic on the care provided to women during labor and delivery identified factors considered unsatisfactory in the care provided to women in labor in relation to good practices, such as hospitalization in the latent phase of labor and low availability of non-pharmacological methods for pain relief and stimulation of breastfeeding in the first hour of a newborn’s life^(30)^.

Finally, this study highlights the high proportion (90.45%) of women with a companion during childbirth. This data demonstrates, in the context of the COVID-19 pandemic, the guarantee of women’s right to a companion, guaranteed by Law 11.108/2005, which guarantees women in labor the presence of a companion of their choice during labor, delivery and the postpartum period in health services within the SUS^(31)^.

The continuous support provided by the companion during labor/delivery is characterized as a protective factor for the physiology of childbirth by favoring the reduction of harmful and aggressive interventions and practices at a time when the postpartum woman and the newborn are vulnerable to obstetric interventions^(32)^.

During the pandemic, many locations in Brazil failed to comply with the Companion Law, citing biosafety, the lack of budget for purchasing personal protective equipment and the atypical moment experienced as justification^(32)^.

It is also important to highlight the role of doulas in supporting women in the obstetric setting, as they not only provide physical and emotional support, but also contribute to a less interventionist care model. They can help develop a birth plan that respects women’s rights and contains their wishes for labor and delivery, and can provide information on possible interventions if necessary^(33)^.

Most of the women who participated in this study (91.82%) were monitored by obstetric nurses. Studies demonstrate the importance of the presence of an obstetric nurse to reduce interventions, given that they use humanized techniques to conduct a respectful birth focused on mother and child well-being. Research has shown that births assisted by obstetric nurses are equipped with a greater number of good practices and have fewer interventions in the physiology of labor and birth, thus emphasizing the importance of the role of this professional in labor and in low-risk vaginal births^(34)^.

The importance of obstetric nurses’ assistance in ensuring useful obstetric interventions, as identified by this study, can be related to the high proportion of use of partographs, non-pharmacological methods for pain relief, and freedom of movement during labor. Furthermore, the care provided by these professionals allowed the use of practices considered ineffective, which medicalize and pathologize the birth process, to be addressed, even in the context of the COVID-19 pandemic, which was evidenced by the low use of oxytocin and episiotomy during deliveries.

In a context of potential worsening of maternal and child indicators, due to the need to reorganize health practices in obstetric care services, the expression of improvement in the reduction of unnecessary interventions stands out, such as the use of episiotomy and the Kristeller maneuver, as well as the lithotomy position and the appropriate use of beneficial technologies, citing the presence of companions, assistance by obstetric nurses, the possibility of walking, and the provision of non-pharmacological and pharmacological methods for pain relief^(35)^.

This reorganization is directly reflected in the improvement of perinatal health and, consequently, in maternal morbidity and mortality rates, the tackling of which is one of the SDGs.

### Study limitations

Despite the methodological rigor adopted and the collection of results in line with the published literature, this study has limitations, which should be considered, especially the fact that the sample is not representative of the city of Belo Horizonte and the Brazilian population, given that it was conducted in hospitals in the city’s public network. It is also worth mentioning that the number of cesarean sections in the private network is much higher than in the public network. Furthermore, the data were collected from medical records made available by the institutions, with potential loss of relevant information. Finally, it was conducted in maternity hospitals with well-established childbirth care models, with effective participation of obstetric nurses, which may interfere with the findings.

### Contributions to nursing, health or public policy

This study contributes significantly to nursing by addressing the best clinical practice of obstetric nursing, assessing the interventions that were carried out during the COVID-19 pandemic, in addition to promoting autonomy for professionals in reducing unnecessary obstetric interventions.

For the health sector, the possibilities of promoting women’s autonomy and quality of life by minimizing the harmful effects of avoidable obstetric interventions stand out.

## CONCLUSIONS

The data from this study demonstrated the existence of distinct profiles of childbirth care in the institutions investigated, which differ in terms of the use of good practices in childbirth care, oscillating between respect for physiology and medicalization. Therefore, it is necessary to improve labor and birth care, aiming to promote women’s autonomy, leading role and well-being. Such aspects, especially in critical epidemiological scenarios, such as COVID-19, can be decisive in the maternal and child outcomes monitored by the SDGs.
