# Supplementary material for: Profiles of obstetric interventions during COVID-19 and advocacy for Sustainable Development Goals
Source: Rev Bras Enferm. 2025 Sep 1;78(Suppl 1):e20240135. doi: 10.1590/0034-7167-2024-0135 (PMC12407857; doi:10.1590/0034-7167-2024-0135)
Supplement: Translation

# Translation: Perfis de intervenções obstétricas durante a COVID-19 e a defesa dos Objetivos de Desenvolvimento Sustentável

**DOI:** 10.1590/0034-7167-2024-0135pt

**Authors:** Thalita Beatriz Santos Maciel, Mery Natali Silva Abreu, Thales Philipe Rodrigues da Silva, Ana Paula Vieira Faria, Rafaela Siqueira Costa Schreck, Carolina Machado Moreira, Bruna Figueiredo Manzo, Fernanda Penido Matozinhos

**Affiliations:** I Universidade Federal de Minas Gerais. Belo Horizonte, Minas Gerais, Brasil; II Universidade Federal de São Paulo. São Paulo, São Paulo, Brasil

**Keywords:** Intervenções Obstétricas, Assistência ao Parto, COVID-19, Enfermagem, Desenvolvimento Sustentável

## Abstract

**Objetivos:**

avaliar os perfis de intervenções obstétricas durante a pandemia da COVID-19 em hospitais públicos de Belo Horizonte, Minas Gerais, Brasil.

**Métodos:**

estudo observacional, com delineamento transversal, aninhado a uma coorte, incluindo 1.717 puérperas. Para identificar os perfis das práticas obstétricas, procedeu-se à técnica de Análise de Componentes Principais.

**Resultados:**

geraram-se perfis divergentes de coexistência de intervenções obstétricas em mulheres com perfis obstétricos análogos. Tendo em vista que as mulheres pertencentes aos perfis formados tenderam a receber o mesmo conjunto de intervenções, concluiu-se que os resultados formaram três tipos de perfil, os quais podem ter sido influenciados pelo contexto da COVID-19.

**Conclusões:**

os achados deste estudo demonstram a necessidade de melhorar a assistência ao trabalho de parto e ao nascimento, visando à promoção da autonomia, do protagonismo e do bem-estar das parturientes. Tal procedimento pode ser determinante nos desfechos materno-infantis monitorados pelo programa Objetivos de Desenvolvimento Sustentável.

## INTRODUÇÃO

Os modelos de assistência ao parto e ao nascimento vêm passando por inúmeras transformações no mundo ao longo dos anos. O parto evoluiu de um momento de exclusiva vivência feminina - realizado em ambiente domiciliar e caracterizado, prioritariamente, como um evento fisiológico - para um processo institucionalizado, reconhecido como um acontecimento patológico, marcado pela medicalização^(1)^.

A medicalização transforma questões não médicas em médicas, impondo a normatização dos corpos e das práticas sociais e sexuais. Essa prática altera aspectos da vida cotidiana do corpo da mulher, como menstruação, gravidez e parto, em propósitos médicos sociais, passando a ser visto como um objeto e um saber exclusivo da medicina^(2)^.

Nos últimos anos a assistência ao trabalho de parto vem passando por modificações importantes no mundo, traduzidas pelo aumento do uso de diversas práticas orientadas para regular e monitorar o processo fisiológico do nascimento^(3)^. Tais práticas teriam por objetivo principal melhorar os resultados perinatais. Todavia, alerta-se que intervenções realizadas sem indicações clínicas comprometem negativamente a experiência do parto pela mulher^(3)^.

Práticas de intervenção no parto e no nascimento - por exemplo, uso do fórceps, cesariana e infusão de medicações do tipo ocitocina - realizadas com indicações clínicas vêm contribuindo para a redução da morbimortalidade materna e neonatal. Entretanto, no rastro desse sucesso, alastrou-se a ideia de estender seus benefícios a todos os partos, para prevenir desfechos negativos, levando ao uso indiscriminado e rotineiro de tais práticas^(4)^.

Na ótica do controle do risco, moldam-se as atitudes intervencionistas da medicalização em relação ao corpo feminino durante a parturição. Os argumentos favoráveis à segurança e à prevenção de complicações configuram-se como instrumentos capazes de legitimar a noção do funcionamento imperfeito do corpo da parturiente, que necessita de práticas e tecnologias preventivas e curativas para evitar desfechos desfavoráveis^(5)^.

No Brasil o aumento das intervenções desnecessárias relaciona-se com a medicalização da assistência obstétrica, levando às altas taxas de cesáreas, sem indicação clínica. Hoje, contrariando a taxa de referência ajustada para a população brasileira, que está entre 25% e 30%, o País possui uma taxa de cesarianas de 55% no sistema público e de 86% no setor privado de assistência à saúde, posicionando-se como um dos líderes mundiais dessa cirurgia^(6)^.

A Organização Mundial de Saúde (OMS), no bojo do documento “Boas práticas na atenção ao parto normal e nascimento”^(7)^, vem recomendando, desde 1996, que o uso das intervenções seja fundamentado em evidências científicas, definindo quatro categorias: a) Práticas claramente úteis e que devem ser estimuladas; b) Práticas claramente prejudiciais, ou ineficazes, e que devem ser eliminadas; c) Práticas usadas de modo inapropriado durante o trabalho de parto; e d) Práticas que necessitam de estudos para comprovação científica.

Em 2018 a OMS atualizou o documento proposto em 1996, passando o orientar-se pelos “Objetivos de Desenvolvimento Sustentável” (ODS), numa perspectiva da saúde da mulher para a redução da mortalidade materna, com foco na adoção de cuidados de maternidade respeitosos, não apenas para evitar as complicações no parto, como também para tornar a experiência de parir positiva, com melhores resultados físicos, mentais e psicológicos, tanto para a mãe quanto para o bebê e a família^(3)^.

Pesquisa realizada no Brasil evidenciou que a assistência medicalizada no momento do parto e do nascimento caracteriza-se, principalmente, pelo representativo número de cirurgias cesarianas e pelo emprego rotineiro de outros tipos de intervenção, como: tricotomia, episiotomia, amniotomia e posição litotômica no nascimento. O estudo também ressaltou que tais intervenções são reconhecidas como práticas claramente prejudiciais, razão pela qual deveriam ser eliminadas na assistência ao parto ou usadas com restrição, na medida em que podem influenciar a prevalência de complicações puerperais^(8)^.

Autores evidenciaram que a atuação das enfermeiras obstétricas ou obstetrizes contribui para a maior adoção de boas práticas assistenciais no momento do parto. As ações de humanização desses profissionais conformam-se como resistência à medicalização do parto, pautadas no enfrentamento das práticas médicas intervencionistas, na defesa da fisiologia do parto e na integralidade do cuidado^(9)^. Adicionalmente, pontua-se que o cuidado da enfermeira obstétrica busca promover relações de encontro com o outro (a mulher) e suporte técnico, o que contribui para a participação das mulheres e a redução das intervenções desnecessárias^(10)^.

A pandemia de COVID-19 produziu no Brasil mudanças no contexto do parto e do nascimento, uma vez que passou a exigir adaptações nas práticas de saúde. Prevenir essa doença passou a ser o objetivo central, já que pouco se conhecia sobre as consequências da infecção em uma gravidez. Nessa perspectiva, o binômio “mãe-filho” pode ter sofrido mais intervenções obstétricas intervenções obstétricas^(11)^.

Devido às diversas alterações fisiológicas presentes na gestação, sobretudo no sistema imunológico e no respiratório, as grávidas foram incluídas no grupo de risco do COVID-19, juntamente com puérperas, idosos e portadores de doenças crônicas, uma vez que apresentam probabilidade de risco mais elevado se forem infectadas^(12)^.

Estudos apresentaram como desfechos obstétricos em decorrência da infecção por COVID-19: elevada taxa de morbidade e mortalidade materna, incluindo parto prematuro, pré-eclâmpsia, descolamento prematuro de placenta, edema pulmonar e a necessidade de ventilação mecânica^(13,14)^.

Este estudo orientou-se pela seguinte questão de pesquisa: “Quais são os perfis das intervenções obstétricas identificados durante a pandemia da COVID-19?”.

## OBJETIVOS

Avaliar os perfis de intervenções obstétricas realizadas durante a pandemia da COVID-19 em hospitais públicos de Belo Horizonte, Minas Gerais, Brasil.

## MÉTODOS

### Aspectos éticos

O estudo foi aprovado pelo Comitê de Ética em Pesquisa da Universidade Federal de Minas Gerais como parte de um estudo maior, “Parto e aleitamento materno em filhos de mães infectadas por SARS-CoV-2”. O consentimento do paciente foi dispensado por tratar-se de coleta de dados realizada com base nos prontuários.

### Desenho, período e local do estudo

Trata-se de estudo observacional com delineamento transversal, aninhado a uma coorte a partir dos dados do estudo “Parto e aleitamento materno em filhos de mães infectadas por SARS-CoV-2”. Realizou-se em três maternidades públicas referências e exclusivas para atendimento do Sistema Único de Saúde (SUS), com a presença de equipe multidisciplinar, incluindo profissionais da enfermagem obstétrica, da cidade de Belo Horizonte - Minas Gerais, Brasil, no período de 2020 a 2022.

Foram adotadas as diretrizes da rede EQUATOR, por meio da ferramenta STROBE (*Strengthening the Reporting of Observational Studies in Epidemiology*).

### População e critérios de inclusão e de exclusão

A amostra foi constituída por meio de sorteio aleatório entre as mulheres que pariram nos respectivos hospitais durante os três meses de alta incidência de COVID-19 (maio, junho e julho) de 2020 no Brasil.

Incluíram-se neste trabalho todas as puérperas com parto hospitalar com gestação única que tiveram como produto de seus conceptos com 22 semanas ou mais e recém-nascidos vivos e com mais de 500 gramas de peso ao nascer admitidas nas três maternidades selecionadas por ocasião do parto.

Para o cálculo amostral, considerou-se o delineamento de estudo de coorte. A distribuição do número de gestantes por maternidade participante respeitou a proporção do número total de nascimentos de cada maternidade selecionada. Elegeram-se 1.717 puérperas para este estudo.

Por se tratar de um estudo transversal aninhado a um coorte, a coleta foi compreendeu três etapas distintas e independentes. A primeira consistiu na coleta em prontuário eletrônico hospitalar retrospectiva; a segunda e a terceira, na coleta telefônica, dividida em duas etapas, a primeira entre 6 e 12 meses pós-parto e a segunda entre 12 e 24 meses pós-parto. As gestantes foram acessadas em horários diversos e o contato telefônico, realizado por pesquisadores capacitados, considerou pelo menos cinco tentativas. Nos casos de negativa das gestantes quanto a sua participação na pesquisa ou de insucesso nas tentativas de contatá-las, elas foram excluídas/substituídas. A amostra da coleta de dados ficou constituída de 480 gestantes que responderam ao contato telefônico durante o processo de coleta de dados. O perfil da amostra apresentou, por essa razão, valores distintos do número amostral para algumas variáveis.

### Protocolo do estudo

Os dados foram coletados nos prontuários das instituições hospitalares durante o período de maior infecção de COVID-19, entre 2020 e 2022, por meio de um questionário semiestruturado adaptado da pesquisa “Nascimento em Belo Horizonte: trabalho de parto e Pesquisa de Nascimento”^(15)^.

As variáveis do estudo foram selecionadas com base em modelo analítico sobre o tema e na atualização de 2018 da diretriz da OMS “Cuidados Intraparto para uma experiência positiva no parto”^(3)^.

As variáveis foram divididas em três categorias (Quadro 1).

**Quadro 1:** Variáveis selecionadas para o estudo

Fatores sociodemográficos e de saúde	Fatores obstétricos	Práticas obstétricas
Idade. Escolaridade. Cor de pele. Trabalho remunerado. Infecção pela COVID-19.	Número de consultas de pré-natal. Primigesta. Idade gestacional no parto. Presença de enfermeira(o) obstétrica(o). Acompanhante durante o parto. Via de nascimento.	Hidratação venosa. Oferta de dieta. Liberdade de movimentação. Uso de partograma. Métodos não farmacológicos para alívio da dor. Enema. Tricotomia. Posição deitada de costas com as pernas levantadas. Manobra de kristeller. Aminiotomia. Infusão de ocitocina. Analgesia. Episiotomia. Punção de acesso venoso periférico. Uso de fórceps.

### Análise dos resultados e estatística

Inicialmente, as variáveis foram apresentadas em frequências absolutas e relativas (%), com os respectivos intervalos de confiança (IC) de 95%, quando categóricas. Em relação às variáveis numéricas, após verificada a assimétrica, com base no teste Shapiro-Wilk, os resultados foram apresentados por meio de mediana e intervalo interquatílico (IIQ).

Para identificar os perfis das práticas obstétricas, procedeu-se à técnica Análise de Componentes Principais (PCA). Trata-se de um método analítico exploratório que condensa as informações contidas nas variáveis originais observadas em um número menor de variáveis, com perda mínima de informações. A PCA é uma técnica estatística de análise multivariada que transforma linearmente um conjunto original de variáveis inicialmente correlacionadasentresi em umconjunto substancialmente menor de variáveis não correlacionadas que contêm a maior parte da informação do conjunto original^(16)^.

As variáveis incluídas no PCA foram: *hidratação venosa, oferta de dieta, liberdade de movimentação, uso de partograma, métodos não farmacológicos para alívio da dor, enema, tricotomia, posição deitada de costas com as pernas levantadas, manobra de Kristeller, aminiotomia, infusão de ocitocina, analgesia, episiotomia, punção de acesso venoso periférico* e *uso de fórceps*.

As variáveis *hidratação venosa, enema, tricotomia, manobra de kristeller, aminiotomia* e *uso de fórceps* não atingiram cargas fatoriais satisfatórias, razão pela qual foram retiradas do modelo. O coeficiente de Kaiser-Mayer-Olkin (KMO) foi estimado como medida de adequação da PCA, com valores entre 0,5 e 1,0, considerados aceitáveis para este índice. Autovalores superiores a 1 foram considerados na definição do número de componentes, com base no gráfico *scree plot*. A estrutura do componente foi obtida a partir dos indicadores que apresentaram cargas fatoriais superiores 0,3 inferiores a -0,3. Fatores com alta carga fatorial (acima de 0,30, com base no critério adotado) em mais de um componente foram considerados naquele componente com maior carga fatorial.

Os dados foram analisados mediante a utilização do software Stata, versão 16.0.

## RESULTADOS

Este estudo avaliou 1.717 pacientes. A mediana da idade foi de 31 anos (IIQ: 26 - 36). A escolaridade mais prevalente foi a de ensino médio completo (59,4%) e a cor da pele predominante autodeclarada foi a parda (57,2%). Por fim, 311 pacientes (64,8%) possuíam um trabalho remunerado e 34 (4,3%) apresentaram algum sinal ou sintoma da COVID-19 (Tabela 1).

**Tabela 1:** Perfil da amostra, Belo Horizonte, Minas Gerais, Brasil, 2023

Variável	Mediana e IIQ^*^			
Idade	31(26 -36 )			
Escolaridade	n	%	IC 95%^**^	
Ensino superior	68	14,1	11,3	17,5
Ensino médio completo	285	59,3	54,9	63,6
Ensino fundamental completo	102	21,2	17,8	25,1
Educação primária completa	25	5,21	3,53	7,60
Cor da pele	n	%	IC 95%	
Branca	88	18,3	15,1	22,0
Preta	102	21,2	17,8	25,1
Parda	276	57,5	53,0	61,8
Amarela	12	2,5	1,4	4,3
Indígena	2	0,4	0,1	1,6
Trabalho remunerado	n	%	IC 95%	
Sim	311	64,7	60,3	68,9
Não	169	35,2	31,0	39,6
Suspeita ou confirmação de COVID				
Não	749	95,6	93,9	96,8
Sim	34	4,3	3,1	6,0

* IIQ - Intervalo interquartílico;

** IC 95% - Intervalo de Confiança de 95%.

Em relação às variáveis gineco-obstétricas, 78,3% das mulheres realizaram 6 ou mais consultas de pré-natais e 62,6% não eram primíparas. A mediana da idade gestacional do parto foi de 39 semanas. A maioria das mulheres teve acompanhamento da enfermagem obstétrica (91,8%) e 90,4% com acompanhante durante o parto. O tipo de trabalho de parto mais observado na amostra foi o espontâneo (51,8%) (Tabela 2).

**Tabela 2:** Caracterização das variáveis gineco-obstétricas, Belo Horizonte, Minas Gerais, Brasil, 2023

	n	%	IC 95%^*^	
Número de consultas pré-natal				
≥6	956	78,3	75,9	80,5
<6	264	21,6	19,4	24,0
Primigesta				
Sim	642	37,3	35,1	39,7
Não	1.075	62,6	60,2	64,8
Idade gestacional do parto^**^	39(38 - 40)			
Presença do profissional enfermeiro obstetra				
Não	104	8,18	6,79	9,82
Sim	1167	91,8	90,1	93,2
Presença de acompanhante durante o parto				
Sim	1259	90,4	88,7	91,8
Não	133	9,5	8,1	11,2
Trabalho de parto				
Espontâneo	746	51,8	49,2	54,4
Induzido sem sucesso - encaminhada a cesárea	173	12,0	10,4	13,8
Induzido com sucesso	450	31,2	28,9	33,7
Não entrou em trabalho de parto - cesárea	69	4,8	3,8	6,0

* IC 95% - Intervalo de confiança de 95%;

** Mediana e Intervalo interquartílico

A Tabela 3 apresenta as estatísticas descritivas sobre as práticas obstétricas utilizadas durante o trabalho de parto e o parto. Em relação às práticas obstétricas consideradas úteis e que devem ser estimuladas, 8,8% tiveram oferecimento de dieta; 91,3%, liberdade de movimentação; 60,50%, uso de partograma durante o trabalho de parto; e 66,3%, uso de métodos não farmacológicos para alívio da dor.

**Tabela 3:** Caracterização das práticas obstétricas durante o parto, Belo Horizonte, Minas Gerais, Brasil, 2023

	n	%	IC 95%^*^	
Práticas obstétricas consideradas úteis e que devem ser estimuladas				
Oferta de dieta				
Não	14	1,1	0,6	1,9
Sim	1.176	98,8	98,0	99,3
Liberdade de movimentação e movimento				
Não	103	8,6	7,1	10,3
Sim	1.091	91,3	89,6	92,8
Uso de partograma				
Não	491	39,5	36,8	42,2
Sim	752	60,5	57,7	63,1
Métodos não farmacológicos para alívio da dor				
Não	406	33,6	30,9	36,3
Sim	802	66,3	63,6	69,0
Práticas obstétricas consideradas ineficazes e que devem ser eliminadas				
Enema				
Não	1145	99,9	99,3	99,9
Sim	1	0,09	0,01	0,61
Tricotomia				
Não	1149	99,6	99,0	99,8
Sim	4	0,3	0,1	0,9
Posição deitada de costas com as pernas levantadas no momento do parto				
Não	1070	92	90,3	93,5
Sim	92	7,9	6,4	9,6
Manobra de Kristeller				
Não	1186	99,3	98,6	99,6
Sim	8	0,6	0,3	1,3
Práticas obstétricas frequentemente usadas de modo inadequado				
Hidratação venosa				
Não	1073	91,4	89,7	92,9
Sim	100	8,5	7,0	10,2
Infusão de ocitocina				
Não	881	71,5	68,9	74
Sim	350	28,4	25,9	31
Analgesia				
Não	887	71,5	69,0	74,0
Sim	352	28,4	25,9	30,9
Episiotomia				
Não	1166	98,3	97,3	98,9
Sim	20	1,6	1,0	2,6
Acesso				
Não	720	62,6	59,7	65,3
Sim	430	37,3	34,6	40,2

* *IC 95% - Intervalo de confiança de 95%.*

Quanto às práticas consideradas ineficazes e que devem ser eliminadas, enema foi observado apenas 0,09% e a tricotomia foi realizada em 0,35%. Em relação à posição do parto, 7,91% pariram em posição de litotomia. A manobra de Kristeller foi utilizada em 0,67% das mulheres.

No que se refere às práticas obstétricas frequentemente usadas de modo inadequado,8,5% receberam hidratação venosa, 28,3% tiveram infusão de ocitocina, 28,4% utilizaram analgesia durante o TP e parto, 98,3% não se submeteram a episiotomia e 62,6% não fizeram uso de acesso venoso periférico (Tabela 3).

A Tabela 4 mostra os resultados da análise de componentes principais. A partir do ponto de corte adotado, com base no *screen plot*, para autovalores superiores a 1 foram extraídos três componentes com uma variância total explicada de 47,73%.

**Tabela 4:** Cargas fatoriais dos componentes da análise de componentes principais. Brasil, 2023

Variável	Componente 1	Componente 2	Componente 3
Oferta de dieta			0,5150
Liberdade de movimentação e movimento			0,5762
Uso de partograma		-0,4753	
Métodos não farmacológicos para alívio da dor		-0,3943	
Posição deitada de costas com as pernas levantadas no momento do parto		0,5836	
Infusão de ocitocina	0,5722		
Analgesia	0,5226		
Episiotomia		0,4399	
Acesso venoso periférico	0,5291	0,2033	
*Eigenvalue*	1,92	1,28	1,08
*Explained variance (%)*	21,39	35,66	47,73
*Overall KMO*	0,623		

*Nota: Para facilitar a compreensão, eliminaram-se as cargas fatoriais inferiores a 0,3 e considerou-se apenas a maior carga, quando apareceia em dois componentes.*

O componente 1 foi composto por mulheres que se submeteram a infusão de ocitocina, fizeram uso de analgesia e utilizaram acesso venoso periférico.

O componente 2 ficou composto por gestantes que não fizeram uso de partograma, não receberam métodos não farmacológicos para alívio da dor, tiveram seus filhos em posição de litotomia e sofreram episiotomia.

O terceiro componente ficou composto por gestantes que tiveram oferta de dieta e liberdade de movimentação durante o TP e o parto. A análise atingiu um KMO satisfatório (acima de 0,5).

## DISCUSSÃO

Este estudo gerou perfis divergentes de coexistência de intervenções obstétricas em mulheres com perfis obstétricos análogos. Tendo em vista que as mulheres enquadradas nos perfis formados tenderam a receber o mesmo conjunto de intervenções, é preciso, primeiramente, entender os benefícios e malefícios de cada intervenção, particularmente a influência de sua coexistência nos resultados obtidos, assim como o contexto de pandemia, considerando a possibilidade de ter impactado a mudança desses perfis identificados na análise dos resultados.

O perfil sociodemográfico da população avaliada caracterizou-se como de mulheres jovens, pardas, que cursaram o ensino fundamental completo e que trabalhavam fora durante o período da pandemia. Sua taxa de infecção foi considerada baixa, com suspeita ou infecção confirmada pela COVID-19 de 2%. No entanto, pode estar subestimado, pois no Brasil o número de testes de COVID-19 era reduzido e havia uma demora importante na divulgação dos resultados. Os testes oferecidos demoravam, em média, sete dias uteis para a emissão do resultado, agravando as possibilidades de criticidade da doença e retardando a estratégia do cuidado no momento certo. Esses eram problemas importantes de acesso ao atendimento adequado da COVID-19 por gestantes e puérperas^(17)^.

Este estudo apurou que a idade média foi de 30,8 anos. Tal achado equipara-se a estudo semelhante realizado na Alemanha, no qual a idade materna média das mulheres que pariram foi de 33 anos. Além disso, a idade em que as mulheres estão parindo nos últimos anos vem aumentando e a proporção de mulheres alemãs que pariram com mais de 35 anos aumentou em 37% em 2018^(18)^.

O número médio de consultas pré-natais identificado neste estudo foi de 7,82, valor compatível com o recomendado pelo Ministério da Saúde, que preconiza que gestantes com risco habitual realizem no mínimo seis consultas de pré-natal (uma no primeiro trimestre da gravidez, duas no segundo e três no terceiro), sendo ideal que a primeira aconteça no primeiro trimestre e que até a 34ª semana sejam realizadas consultas mensais^(19)^.

A consulta de pré-natal é de extrema importância para informar as mulheres sobre o processo de trabalho de parto e de parto, devendo fornecer informações que permitam escolhas autônomas, para que, munidas de informações, estejam menos vulneráveis às intervenções. No Brasil, no âmbito do Sistema Único de Saúde (SUS), destaca-se a Rede Cegonha, com a proposição de ações estruturadas a partir de quatro componentes: pré-natal; parto e nascimento; puerpério e atenção integral à saúde da criança; e sistema logístico, que se refere ao transporte sanitário e regulação. Nessa conjuntura, o eixo pré-natal é ressaltado como elemento fundamental para a garantia de um atendimento de qualidade, seguro e humanizado para todas as mulheres^(20)^, incluindo os momentos epidemiológicos críticos, como o da COVID-19.

O primeiro perfil, formado por intervenções frequentemente utilizadas de forma inadequada na assistência ao trabalho de parto, inclui parturientes que se submeteram a infusão de ocitocina, fizeram uso de analgesia e punção de acesso venoso periférico e foram submetidas a episiotomia.

As intervenções realizadas durante a assistência ao trabalho de parto e ao nascimento devem ocorrer de forma criteriosa e em situações com indicação clínica de real necessidade. No entanto, tais intervenções ocorrem, muitas vezes, arbitrariamente, atingindo muitas mulheres que são assistidas nos hospitais do País, incluindo o uso indiscriminado de ocitocina^(3)^. A ocitocina é um hormônio produzido pelo hipotálamo e armazenado na hipófise posterior, cuja ação é central no trabalho de parto, pois é responsável pelo estímulo das contrações uterinas^(21)^. Ainda que não seja recomendado pela Organização Mundial da Saúde (OMS), compondo o grupo de práticas que são utilizadas de forma inadequada na assistência obstétrica, a ocitocina sintética é utilizada de forma rotineira em muitas maternidades brasileiras, com o objetivo de aumentar e acelerar as contrações^(21)^. Os dados encontrados por esta pesquisa revelaram o uso de ocitocina para 28,43% das mulheres assistidas nas instituições investigadas, valor considerável, mas abaixo da média nacional de 40% para administração deste hormônio entre as parturientes brasileiras^(22)^.

A prática da episiotomia é uma intervenção obstétrica que consiste em um corte realizado na região perineal, o qual amplia a área de saída do bebê, acelerando o processo de nascimento, não devendo ser utilizada de forma usual^(3)^ Por constituir-se um ato cirúrgico, o procedimento deve ser informado e autorizado pela mulher antes de sua realização, devendo ser apontados os possíveis riscos e benefícios. Essa intervenção vem sendo utilizada, muitas vezes, sem a informação adequada para as mulheres, de forma rotineira, em grande parte das instituições brasileiras, apesar de sua recomendação contemplar apenas 10 a 15% dos casos^(23)^. No entanto, esta pesquisa identificou que 1,69% das mulheres foi submetida à episiotomia durante a assistência ao parto, desvelando, portanto, um cenário mais próximo daquele recomendado pela OMS para que tal prática seja desestimulada^(3)^.

Este estudo concluiu que a punção venosa atingiu 37% das parturientes. A punção venosa, na maior parte dos casos, está associada ao uso de ocitocina sintética para indução e à aceleração ou correção de alterações na evolução do trabalho de parto, sendo considerada, na maioria das vezes, uma intervenção desnecessária^(24)^.

O segundo perfil deste trabalho levou em consideração o uso de práticas obstétricas evidentemente prejudiciais, devendo ser abolidas na assistência obstétrica, considerando mulheres que não utilizaram partograma, não receberam métodos não farmacológicos para alívio da dor e pariram na posição de litotomia.

O partograma é uma ferramenta desenvolvida para melhorar a comunicação entre mulheres e profissionais da saúde. Identificou-se que 60,50% das mulheres das instituições investigadas fizeram uso de partograma durante o trabalho de parto, proporção superior à encontrada na literatura^(25)^, aspecto que demonstra a necessidade de alcançar melhor adesão ao uso do partograma na assistência obstétrica pelos profissionais de saúde. O uso desta ferramenta compõe o perfil das práticas que são úteis e beneficiam a mulher no processo de parto.

O uso de analgesia durante o processo de parto é comum e apresenta benefícios para a parturiente, tornando o processo de parir menos doloroso. Por isso, ainda que a analgesia seja caracterizada como prática utilizada de forma inadequada, no parto vaginal sua utilização deve ser garantida e disponibilizada às mulheres o que solicitarem, com avaliação e monitoramento do bem-estar materno e fetal. Existem opções de analgesia local e métodos não farmacológicos para alívio da dor. Um estudo apurou que a utilização de analgesia local foi menor em proporções de gestantes que tiveram o acompanhamento de profissionais da Enfermagem Obstétrica^(26)^.

Os métodos não farmacológicos constituem uma opção para substituir a analgesia durante o trabalho de parto e o parto e para auxiliar as parturientes a lidar com suas queixas álgicas^(27)^. Este estudo identificou que os métodos não farmacológicos de alívio da dor foram utilizados por 66,39% das mulheres, citando-se entre aqueles mais utilizados, baseados na literatura: banho de chuveiro, banho de banheira, massagem, uso da bola, banquinho e cavalinho. Outro estudo evidenciou que a maior parte das mulheres que utilizaram métodos não farmacológicos para alívio da dor evoluíram para partos vaginais^(28)^, contribuindo para a redução da ansiedade e da dor, transtornos elencados como os grandes responsáveis pelo aumento do número de cesáreas eletivas^(29)^.

O terceiro perfil evidenciou práticas que são úteis e beneficiam a mulher no processo de parto, considerando parturientes que tiveram livre oferecimento de dieta, liberdade de movimento. Esta pesquisa mostrou que 91,37% das mulheres tiveram liberdade de movimentação durante o processo de parturição, que, durante o trabalho de parto, auxilia na progressão do trabalho de parto, ajuda a enfrentar a dor e diminui o risco de episiotomia^(28)^, razão pela qual é indicada como prática obstétrica útil e que deve ser recomendada^(3)^. Durante a pandemia a maioria das posições de parto foi estimulada, dando preferência às verticalizadas. Porém, houve orientação de que o parto na água deveria ser evitado, devido à presença de vírus nas fezes, com elevado potencial de contaminação^(29)^.

Estudo qualitativo realizado com o propósito de avaliar as repercussões da pandemia na atenção à mulher no trabalho de parto e parto identificou fatores considerados insatisfatórios na assistência às parturientes em relação às boas práticas, como: internação em fase latente do trabalho de parto e baixa oferta dos métodos não farmacológicos para o alívio da dor e de estímulo ao aleitamento materno na primeira hora de vida do recém-nascido^(30)^.

Por fim, destaca-se neste estudo a proporção elevada - 90,45% - das mulheres com acompanhante durante o parto. Esse dado demonstra no contexto da pandemia da COVID-19 a garantia do direito das mulheres ao acompanhante, assegurado pela Lei 11.108/2005, que garante às parturientes a presença de um acompanhante de sua escolha durante o trabalho de parto, parto e puerpério em serviços de saúde no âmbito do SUS^(31)^.

O apoio contínuo proporcionado pelo acompanhante durante o trabalho de parto/parto caracteriza-se como fator protetor à fisiologia do parto ao favorecer a redução de intervenções e práticas danosas e agressivas em um momento em que a parturiente e o recém-nascido encontram-se vulneráveis às intervenções obstétricas^(32)^.

No período da pandemia ocorreu em muitas localidades do Brasil o descumprimento da Lei do Acompanhante, citando-se como justificativa a biossegurança, a falta de orçamento para compras de equipamentos de proteção individual e o momento atípico vivenciado^(32)^.

Deve-se destacar, também, o papel das doulas no acompanhamento às mulheres no cenário obstétrico, as quais, além de proporcionarem apoio físico e emocional, contribuem para um modelo de assistência menos intervencionista. Elas podem auxiliar na elaboração de um plano de parto submetido aos direitos e contendo os desejos das mulheres para o trabalho de parto e o parto, podendo, se necessário, fornecer informações sobre possíveis intervenções^(33)^.

A maioria das mulheres que compuseram esta pesquisa (91,82%) teve acompanhamento de profissionais da enfermagem obstétrica. Estudos demonstram a importância da presença da enfermeira obstetra para a diminuição de intervenções, tendo em vista que ela utiliza técnicas humanizadas para a condução de um parto respeitoso e focado no bem-estar do binômio mãe-filho. Pesquisa evidenciou que os partos assistidos pela Enfermagem Obstétrica são dotados de maior quantidade de boas práticas e possuem menos intervenções à fisiologia do parto e do nascimento, enfatizando, assim, a importância da atuação dessa profissional no trabalho de parto e no parto vaginal de risco habitual^(34)^.

É possível relacionar a importância da assistência das enfermeiras obstétricas para a garantia das intervenções obstétricas consideradas úteis, conforme identificado por este estudo com a alta proporção de uso do partograma, dos métodos não farmacológicos para alívio da dor e liberdade de movimentação no trabalho de parto. Ademais, o cuidado prestado por essas profissionais permitiu o enfrentamento ao emprego das práticas consideradas ineficazes, que medicalizam e patologizam o processo de nascimento, ainda que no contexto da pandemia de COVID-19, o que foi evidenciado pelo baixo uso da ocitocina e da episiotomia nos partos.

Em um contexto de potencial piora dos indicadores materno-infantis, em decorrência da necessidade de reorganizar as práticas de saúde nos serviços de atenção obstétrica, destaca-se a expressão de melhoria à redução de intervenções desnecessárias, como o uso de episiotomia e da manobra de Kristeller, assim como da posição de litotomia e do uso apropriado de tecnologias benéficas, citando-se a presença de acompanhantes, a assistência por enfermeiras obstetras, a possibilidade de deambulação, e a oferta de métodos não farmacológicos e farmacológicos para alívio da dor^(35)^.

Tal reorganização reflete-se diretamente na melhoria da saúde perinatal e, consequentemente, nas taxas de morbimortalidade materna, cujo enfrentamento compõe um dos Objetivos de Desenvolvimento Sustentável (ODS).

### Limitações do estudo

Apesar do rigor metodológico adotado e da apuração de resultados consonantes com a literatura publicada, este estudo apresenta limitações, as quais devem ser consideradas, destacadamente o fato de a amostra não ser representativa da cidade de Belo Horizonte e da população brasileira, tendo em vista que foi realizado em hospitais da rede pública do município. Cita-se, ainda, que na rede privada o número de cesarianas é muito superior àquele da rede pública. Ademais, os dados foram coletados em prontuários disponibilizados pelas instituições, com potencial perda de informações relevantes. Por fim, foi realizado em maternidades com modelos de assistência ao parto já bem implantados, com atuação efetiva de enfermeiras obstétricas, o que pode interferir nos achados.

### Contribuições para a área da Enfermagem

Este estudo contribui de forma significativa para o campo da Enfermagem ao abordar a melhor prática clínica da enfermagem obstétrica, avaliando as intervenções que foram realizadas no período da pandemia da COVID-19, além de impulsionar a autonomia dos profissionais na diminuição de intervenções obstétricas desnecessárias.

Para o setor da Saúde destacam-se as possibilidades de promover a autonomia da mulher e a qualidade de vida ao minimizar os efeitos deletérios das intervenções obstétricas evitáveis.

## CONCLUSÕES

Os dados referentes a este estudo demonstraram a existência de distintos perfis de assistência ao parto nas instituições investigadas, que divergem quanto ao uso das boas práticas da assistência ao parto e ao nascimento, oscilando entre o respeito à fisiologia e a medicalização. Portanto, faz-se necessário melhorar a assistência ao trabalho de parto e ao nascimento, visando à promoção da autonomia, do protagonismo e do bem-estar das mulheres. Tais aspectos, especialmente em cenários epidemiológicos críticos, como o da Covid-19, podem ser determinantes nos desfechos materno-infantis monitorados pelos ODS.
